# Iron Metabolism as a Potential Mechanism for Inducing TRAIL-Mediated Extrinsic Apoptosis Using Methylsulfonylmethane in Embryonic Cancer Stem Cells

**DOI:** 10.3390/cells10112847

**Published:** 2021-10-22

**Authors:** Nipin Sp, Dong Young Kang, Eun Seong Jo, Jin-Moo Lee, Kyoung-Jin Jang

**Affiliations:** 1 Department of Pathology, Institute of Biomedical Science and Technology, School of Medicine, Konkuk University, Chungju 27478, Korea; nipinsp@konkuk.ac.kr (N.S.); kdy6459@kku.ac.kr (D.Y.K.); 2 Pharmacological Research Division, National Institute of Food and Drug Safety Evaluation, Osong Health Technology Administration Complex, Cheongju-si 28159, Korea; eses0706@korea.kr (E.S.J.); elzemy@gmail.com (J.-M.L.); 3 SK Bioscience, Seongnam-si 13493, Korea

**Keywords:** NCCIT, NTERA-2, MSM, TRAIL, iron metabolism, p38/p53/ERK signaling, miR-130a/miR-221/miR-222

## Abstract

Embryonic cancer stem cells (CSCs) can differentiate into any cancer type. Targeting CSC using natural compounds is a good approach as it suppresses cancer recurrence with fewer adverse effects, and methylsulfonylmethane (MSM) is a sulfur-containing compound with well-known anticancer activities. This study determined the mechanistic aspects of the anticancer activity of MSM. We used Western blotting and real-time qPCR for molecular signaling studies and conducted flow cytometry for analyzing the processes in cells. Our results suggested an inhibition in the expression of CSC markers and Wnt/β-catenin signaling. MSM induced TRAIL-mediated extrinsic apoptosis in NCCIT and NTERA-2 cells rather than an intrinsic pathway. Inhibition of iron metabolism-dependent reactive oxygen species (ROS) generation takes part in TRAIL-mediated apoptosis induction by MSM. Suppressing iron metabolism by MSM also regulated p38/p53/ERK signaling and microRNA expressions, such as upregulating miR-130a and downregulating miR-221 and miR-222, which resulted in TRAIL induction and thereby extrinsic pathway of apoptosis. Hence, MSM could be a good candidate for neoadjuvant therapy by targeting CSCs by inhibiting iron metabolism.

## 1. Introduction

Embryonic stem (ES) cells can differentiate into all derivatives of embryonic germ layers, ectoderm, endoderm, and mesoderm, whereas embryonic cancer stem cells (CSCs) have these abilities along with uncontrolled proliferation, making them more dangerous than other cancer cells [1,2]. ES cells can generate all kinds of tissues and are derived from the inner cell mass of the blastocyst [3]. Hence, embryonic CSC could differentiate into various cancers, such as lung cancer, breast cancer, and colon cancer [4]. Many signaling pathways could promote pluripotency and self-renewal ability of CSC, and among them, the Wnt/β-catenin pathway is considered a vital signaling pathway in CSC tumor progression [5,6]. In canonical Wnt signaling, secreted glycoprotein Wnt family proteins along with β-catenin, a transcription activator factor for the Wnt family, promote homeostasis and embryonic development [7]. The activation of β-catenin was regulated by casein kinase 1α and glycogen synthase kinase 3β (GSK-3β) for ubiquitination or proteasomal degradation. The development continues when β-catenin helps in transcription by binding to transcription factor (TCF)-binding element in TCF/lymphoid enhancer-binding factor (LEF) in the nucleus [8]. Along with Wnt/β-catenin signaling, CSC markers also play a key role in developing CSC. Sex-determining region Y (SRY)-box 2 (SOX2), octamer-binding transcription factor 4 (OCT4), and homeobox protein NANOG were overexpressed in CSC, helping to promote tumorigenesis, and they can maintain the pluripotent nature of CSC [9,10]. Hence, targeting these Wnt/β-catenin signaling and CSC markers is an effective treatment method against CSC and targets cancer cells [11,12].

Targeting apoptosis induction in CSC instead of cancer cells is a better way to suppress tumor progression. Tumor necrosis factor-related apoptosis-inducing ligand (TRAIL) is a death ligand and induces extrinsic apoptosis pathway by binding to trimeric death receptors and activate their signaling mechanism [13]. DR4 or DR5 receive signals from TRAIL and transfer the signals toward a cytoplasmic death domain-containing adaptor protein, tumor necrosis factor receptor-associated death domain (TRADD) protein [14]. These signals activate caspase-8 or -10 and initiate extrinsic apoptosis by cleaving the executioner caspase-3, -6, or -7 [15,16]. Hence, the TRAIL-mediated extrinsic apoptosis pathway is considered a therapeutic target for chemotherapy, and induction of TRAIL sensitivity defines the efficacy of a chemotherapeutic drug [4,17].

Iron is a vital component in hemoglobin, and it takes part in cellular metabolism through its enzymatic activities in cellular functions by regulating the factors responsible for cell proliferation and death. It also mediates DNA synthesis and damage response or repair and mediates mitochondrial functions [18]. Iron is an essential element in the body, and it plays a crucial role in oxygen transport. Moreover, reactive oxygen species (ROS) generation occasionally occurs during biomolecule oxidation that makes iron toxic [19]. Preventing excessive ROS generation could be controlled through iron homeostasis, where iron transport occurs with the help of iron channels. Furthermore, iron could play an important role in cancer by maintaining the genomic stability and regulation of epigenetic alterations. Iron also mediates tumor microenvironment and tumor metastasis [20]; possessing the dual role of tumor growth and cancer cell death depends on the condition it takes part in cellular functions [21]. The M1 and M2 macrophages also take part in iron metabolism as the M1 macrophages strive for iron accumulation by heme oxygenase 1 (HO-1) and ferroportin (FPN), while M2 macrophages, along with ferritin, reduce the amount of iron by exporting and suppressing the pro-inflammatory cytokines [22]. This shows the importance of iron metabolism in the body, as their alteration may lead to inflammation and tumorigenesis.

Using the natural compound for cancer treatment is a good approach, as it provides an option of multi-targeted treatment with fewer side effects than commercially available chemotherapeutic drugs [23,24]. Methylsulfonylmethane (MSM) is a sulfur-containing compound that occurs naturally in animals, plants, and other natural compounds and foods, including fruits, vegetables, and beverages [25]. This compound exerts various physiological activities, including antioxidant and anti-inflammatory activities [26,27]. MSM is well known for its anticancer activity against various cancer types, including breast [28], lung [29], colorectal [30], and gingival cancers [31]. This MSM can also synergize chemotherapeutic drug activity to increase efficacy and reduce the adverse effect caused by the chemotherapeutic drug [32,33]. The U.S. Food and Drug Administration classified MSM as generally recognized as a safe grade molecule [34]. As MSM is well known for its anti-cancer ability, its activity against embryonic cancer cells would be interesting if it could target CSCs.

This study demonstrated the effect of MSM in iron metabolism and its role in TRAIL-mediated extrinsic apoptosis in NCCIT and NTERA-2 embryonic CSCs. We also analyzed the molecular mechanism behind the apoptosis induction of MSM in CSCs.

## 2. Materials and Methods

### 2.1. Antibodies and Cell Culture Reagents

Roswell Park Memorial Institute-1640 (RPMI-1640) medium, penicillin–streptomycin solution, and trypsin-EDTA (0.05%) were purchased from Gibco (Thermo Fisher Scientific, Inc., Waltham, MA, USA). Dulbecco’s modified Eagle’s media (DMEM; LM001-51) was purchased from Welgene Biotech (Taipei City, Taiwan). MSM (PHR1346); fetal bovine serum (FBS; 12003C); and primary antibodies specific for SOX2 (MAB4423), OCT4 (MABD76), NANOG (MABD24), SB 203580 (S8307), PD 98,059 (P215), and iron (II) sulfate heptahydrate (F8633) were purchased from Sigma-Aldrich (Merck KGaA, St. Louis, MO, USA). Antibodies specific for β-actin (sc-47778), TRADD (sc-46653), Wnt5A (sc-365370), Casp8 (sc-81656), Bcl-2 (sc-7382), p21 (sc-756), cyclin E (sc-481), and CDK4 (sc-260), and secondary antibodies (anti-mouse (sc-516102) and anti-rabbit (sc-2357)) were obtained from Santa Cruz Biotechnology, Inc. (Dallas, TX, USA). The Wnt8A (H00007478-B01P) antibody was obtained from Abnova (Taipei City, Taiwan), and β-Catenin (#9582), GSK-3β (#9315), Bax (#2772), Casp3 (#9662), C-Casp3 (#9661), cytochrome C (#11940), p27 Kip1 (#3686), p53 (#9282), pATM (#5883), pATR (#2853), pCHK1 (#2348), pCHK2 (#2197), pBRCA1 (#9009), p38 (#8690), pp38 (#4511), ERK (#4695), and pERK (#4370) antibodies and a TCF/LEF Family Antibody Sampler Kit (#9383) were purchased from Cell Signaling Technology, Inc. (Beverly, MA, USA). TRAIL (ab42121), DR4 (ab8415), DR5 (ab8416), TFR1 (ab84036), STEAP3 (ab151566), DMT1 (ab55735), and cyclin D1 (ab6152) antibodies were purchased from Abcam (Cambridge, MA, USA). Finally, FPN1 (NBP1-21502) and iNOS (NB300-650) antibodies were obtained from Novus Biologicals (Littleton, CO, USA).

### 2.2. Cell Culture and Treatment

NCCIT (CRL-2073) and NTERA-2 (CRL-1973) cell lines were purchased from the American Type Culture Collection (ATCC; Manassas, VA, USA). NCCIT cells were cultured and maintained in RPMI-1640 media, and NTERA-2 cells were cultured and maintained in DMEM media plus 10% FBS and 1% penicillin at 37 °C in 5% CO_2_. Subsequently, the medium was changed three times a week after cells were grown for up to 80% confluence and treated with MSM. Then, the setup was incubated at 37 °C for 48 h.

### 2.3. Cell Viability Assay

Cell viability was measured using 3-(4,5-dimethylthiazol-2-yl)-2,5-diphenyltetrazolium bromide (MTT) assay. NCCIT or NTERA-2 cells were maintained in culture media in 96-well culture plates at 3 × 10^3^ per well (density) for 24 h. Next, cells were incubated on a new medium containing dimethyl sulfoxide (DMSO) as the vehicle control and then treated with various concentrations of MSM (50–400 mM) for 48 h. Subsequently, MTT (5 mg/mL) was added and the mixture was incubated for 4 h at 37 °C. The resulting formazan product was then dissolved in DMSO, after which an Ultra Multifunctional Microplate Reader (Tecan, Durham, NC, USA) was used to measure the absorbance at a wavelength of 590 nm. All measurements and experiments were conducted in triplicate.

### 2.4. Western Blotting

Protein samples were isolated from untreated (control) or MSM-treated, p38 inhibitor treated (1 h), or ERK inhibitor treated (1 h), or FeSO_4_ treated (48 h) NCCIT or NTERA-2 cells using a radioimmunoprecipitation (RIPA) lysis buffer (20–188; EMD Millipore), containing protease and phosphatase inhibitors. First, the concentration of proteins was measured using Bradford’s method (Thermo Fisher Scientific). Next, the same amounts of isolated protein samples (100 μg/well) were resolved with sodium dodecyl sulfate-polyacrylamide gel (10–15%) electrophoresis. The separated proteins were then transferred onto nitrocellulose membranes, followed by blocking with 5% skim milk (BD Biosciences, San Jose, CA, USA) in TBS-T buffer (20-mM Tris-HCl (Sigma-Aldrich; Merck KGa A, pH 7.6), 137-mM NaCl (Formedium, Norfolk, UK; NAC03), and 0.1× Tween20 (Scientific Sales, Inc., OakRidge, TN, USA)) for 1 h. Next, the membranes were incubated overnight at 4 °C in a shaker with specific primary antibodies diluted in 5% bovine serum albumin (EMDMillipore). Then, membranes were washed with TBS-T and incubated with HRP-conjugated secondary antibodies for 1 h at room temperature. Finally, the detection was performed using a Femto Clean Enhanced Chemiluminescence Solution Kit (77449; GenDEPOT, Katy, TX, USA) in a LAS-4000 imaging device (Fujifilm, Tokyo, Japan). Quantifications were conducted using ImageJ software (v.1.8.0_172; National Institutes of Health).

### 2.5. Quantitative Real-Time Polymerase Chain Reaction (qPCR)

Total RNA was isolated using the RNeasy Mini Kit (Qiagen GmbH, Hilden, Germany) and then quantified using a spectrophotometer at 260 nm. Subsequently, a thermal cycler (C1000 Thermal Cycler; Bio-Rad, Hercules, CA, USA) was used to make cDNA from the total RNA using a first-strand cDNA synthesis kit (Bioneer, Daejeon, Korea) and oligo (dT) primers. PD-L1, p53, and GAPDH cDNA were amplified using an RT-PCR Premix Kit (Bioneer) with primers synthesized by Bioneer. Furthermore, the Light Cycler 480II (Roche) was used for qPCR as follows: 2 μL diluted cDNA was mixed with 10 μL TB Green Advantage Premix (Takara Bio, Ōtsu, Japan) and 1 μL each of forward and reverse primers. The cycling conditions were as follows: 95 °C for 5 min for the initial denaturation, followed by 40 cycles of 95 °C for 40 s, 58 °C for 40 s, 72 °C for 40 s, and finally extension for 5 min at 72 °C. All reactions were conducted three times and normalized to GAPDH, and quantifications were conducted using obtained Cp values.

### 2.6. Fluorescence-Activated Cell Sorting (FACS) Analysis for Mitochondrial and Cellular ROS

After the cultured cells were washed with pre-warmed culturing medium supplemented with 10% FBS (staining buffer), 1 × 10^6^ cells were re-suspended in 1 mL staining buffer containing MitoSOX (5 μM; M36008; Invitrogen, Carlsbad, CA, USA) for mROS or CM-H2DCFDA (5 μM; Invitrogen, C6827) for cellular ROS. Then, the cells were incubated in a CO_2_ incubator at 37 °C for 30 min. Finally, the stained cells were washed with 1 mL pre-warmed staining buffer and used for FACS analysis. The analysis was performed using FlowJo software.

### 2.7. Cell Cycle Analysis

The DNA content of MSM-treated and non-treated cells was determined using a BD Cycletest Plus DNA Reagent Kit (BD Biosciences, San Jose, CA, USA) according to the manufacturer’s protocol. Approximately 5 × 10^5^ cells, with or without MSM for 48 h, were washed with PBS and permeabilized with trypsin. The neutralization of RNA interaction with propidium iodide (PI) was performed by treating the cells with RNase buffer and trypsin inhibitor. The samples were then stained with PI and incubated for 30 min in the dark at room temperature and analyzed by a FACSCalibur flow cytometer (BD Biosciences, San Jose, CA, USA).

### 2.8. Comet Assay

The comet assay kit (Abcam, Cambridge, MA, USA) was used for measuring the cellular DNA damage. This assay is a single-cell gel electrophoresis method for a simple evaluation of cellular DNA damage. First, a base layer of comet agarose was created on a slide, and then a layer of cells and agarose was added, followed by lysis. Next, electrophoresis was performed under neutral conditions, and the cells were stained with DNA dye. Finally, cell morphology was observed by fluorescence microscopy (Olympus IX71/DP72).

### 2.9. Apoptosis Analysis

Fluorescein-conjugated annexin V (annexin V-FITC) was used to measure apoptosis in NCCIT and NTERA-2 cells. First, the MSM-treated or untreated cells were washed with PBS and re-suspended in a binding buffer at a concentration of 1 × 10^6^ cells. Then, the cells were stained with annexin V-FITC and PI for 10 min in a dark room at room temperature. Finally, the percentage of apoptotic cells was measured by flow cytometry via FACSCalibur, and the analysis was performed using FlowJo software.

### 2.10. Human TRAIL Enzyme-Linked Immunosorbent Assay (ELISA)

This method was performed by ELISA for quantitative detection using a Human TRAIL ELISA Kit from Invitrogen (BMS2004; Carlsbad, CA, USA). The NCCIT or NTERA-2 cells were treated with 200-mM MSM for 48 h, and spent media were used for the assay. The samples were added to anti-human TRAIL-coated micro-wells along with sample diluent and a biotin-conjugated solution. After incubation, streptavidin-HRP was added and further incubated; 3,3′,5,5′-tetramethylbenzidine (TMB) solution was added after washing. Finally, a stop solution was added once the high-concentrated standard turned dark blue. The absorbance was read at 450 nm, and calculations were performed according to assay protocol.

### 2.11. Caspase-Glo 3/7 Assay

This method was performed using the Caspase-Glo 3/7 Assay System from Promega (G8090; Fitchburg, WI, USA). The NCCIT or NTERA-2 cells were seeded (20,000 cells per well) in a white-walled 96-well plate and treated with MSM after reaching 70–80% confluence. After incubation with MSM, Caspase-Glo 3/7 Reagent was added to each well and incubated in a plate shaker at 500 rpm for 3 h. After incubation, readings were taken using a plate-reading luminometer, and calculations were done according to assay protocol.

### 2.12. Iron Assay Analysis

Iron estimation was performed using an iron assay kit (MAK025) purchased from Sigma-Aldrich (Merck KGaA, St. Louis, MO, USA). Briefly, NCCIT or NTERA-2 cells (2 × 10^6^) treated with or without MSM were homogenized in an iron assay buffer and collected by centrifugation at 16,000× *g* for 10 min at 4 °C. Then, the lysates were mixed with an iron assay buffer, added to a 96-well plate along with an iron reducer, and incubated in a horizontal shaker for 30 min at 25 °C. Then, 100 µL of the iron probe was added to each well and incubated for 1 h at 25 °C. Absorbance was measured at 593 nm, and controls were set to 100% for comparison.

### 2.13. FACS Analysis for Ferrous Ion (Fe^2+^)

After cultured cells (NCCIT or NTERA-2) were washed with culturing medium, cells were stained in 2 mL of the staining solution containing FerroFarRed (5 µM; GC903-01; GORYO Chemical, Sapporo, Japan) and incubated in a CO_2_ incubator at 37 °C for 30–40 min. After staining, cells were washed with 1 mL of pre-warmed serum-free culture medium and used for fluorescence-activated cell sorting (FACS) analysis.

### 2.14. Transfections of miRNA Inhibitor

NCCIT and NTERA-2 cells (1 × 10^5^ cells) were seeded in six-well plates and grown up to 60% confluence. The cells were then transfected with anti-miR (microRNA inhibitor; AM 17,000; Thermo Fisher Scientific, Inc., Waltham, MA, USA) using a Lipofectamine transfection reagent (Thermo Fisher Scientific, Inc., Waltham, MA, USA) for 24 h. Then, cells were treated with/without MSM for 48 h, and then total RNA was isolated to analyze using real-time qPCR.

### 2.15. Statistical Analyses

All experiments were performed in triplicate. The results were expressed as the mean ± standard error of the mean. Statistical analyses were conducted via one-way analysis of variance (ANOVA) or Student’s *t*-test. Additionally, one-way ANOVA was performed using Tukey’s post hoc test. The analyses were performed using SAS 9.3 software (SAS Institute, Inc., Cary, NC, USA). A *p*-value < 0.05 (*) was considered to indicate a significant difference.

## 3. Results

### 3.1. MSM Inhibited CSC Markers and Wnt/β-Catenin Signaling in Embryonic CSCs

**Figure 1:**
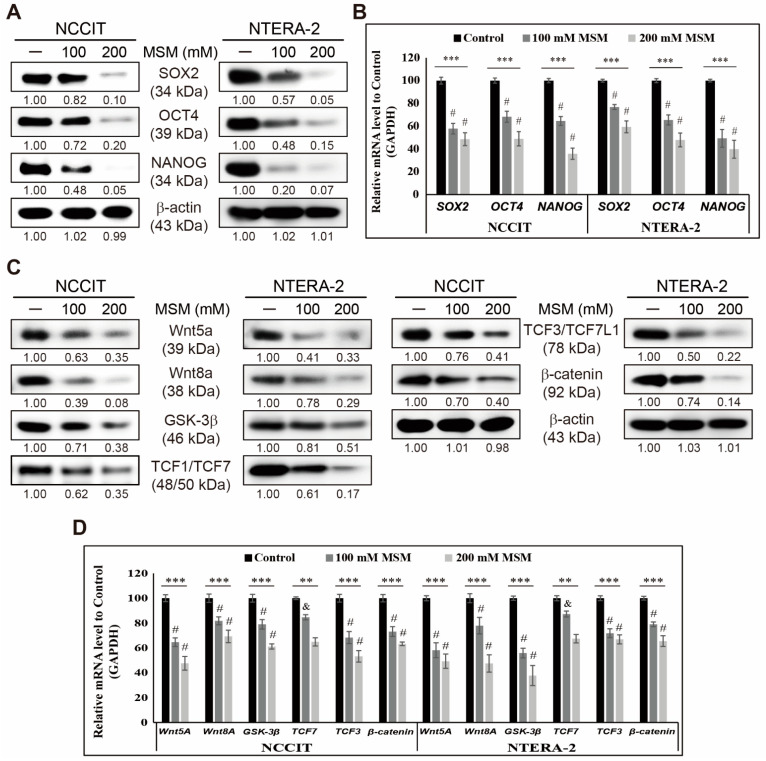
MSM inhibits CSC markers and Wnt/β-catenin signaling. (**A**) Western blotting of SOX2, OCT4, and NANOG proteins in NCCIT and NTERA-2 cells after treatment with 100 and 200 mM MSM for 48 h. Expression levels were estimated by densitometry and normalized to β-actin. Data were obtained in triplicate. (**B**) Real-time qPCR showing illustrative expression of SOX2, OCT4, and NANOG genes in NCCIT and NTERA-2 cells. Cp values were normalized to GAPDH mRNA. Controls were set at 100. *** p < 0.001 (ANOVA). *# p* < 0.001 vs. control. (**C**) Western blotting of Wnt5a, Wnt8A, GSK-3β, β-catenin, TCF1/TCF7, and TCF3/TCF7L1 proteins in NCCIT and NTERA-2 cells after treatment with 100 and 200 mM MSM for 48 h. Expression levels of proteins were estimated by densitometry and normalized to β-actin. Experiments were conducted in triplicate. (**D**) Real-time qPCR showing illustrative expression of Wnt5a, Wnt8A, GSK-3β, β-catenin, TCF7, and TCF3 genes in NCCIT and NTERA-2 cells. Cp values were normalized to GAPDH mRNA. Controls were set at 100. ** p < 0.01 (ANOVA). & *p* < 0.01 vs. control. *** p < 0.001 (ANOVA). # *p* < 0.001 vs. control.

First, we analyzed the effect of MSM on embryonic CSC viability by MTT assay. We used increasing concentrations of MSM in NCCIT (Supplementary Figure S1A) and NTERA-2 (Supplementary Figure S1B) and found a concentration-dependent inhibition in cell viability by MSM. From these two cell lines, we used 100 and 200 mM MSM for further experiments. Then, we checked whether MSM could inhibit CSC markers in embryonic CSCs. Analysis of CSC markers SOX2, OCT4, and NANOG in NCCIT, and NTERA-2 cells upon MSM treatment, showed downregulation in the expression patterns of CSC markers (Figure 1A). We confirmed the inhibition of CSC markers by MSM at the mRNA level using real-time qPCR analysis (Figure 1B). These results suggested the ability of MSM to inhibit CSC proliferation. Then, we analyzed the expression patterns of Wnt/β-catenin signaling in embryonic CSC in the presence of MSM. The obtained results indicated an inhibition in the expression levels of Wnt5a, Wnt8A, GSK-3β, β-catenin, and TCF proteins by MSM treatment in NCCIT and NTERA-2 cells (Figure 1C). We then confirmed the inhibition of Wnt/β-catenin signaling in mRNA level by MSM treatment in embryonic CSC (Figure 1D). These results indicated the capability of MSM against CSCs.

### 3.2. MSM Induced ROS and DNA Damage Response in Embryonic CSC

**Figure 2:**
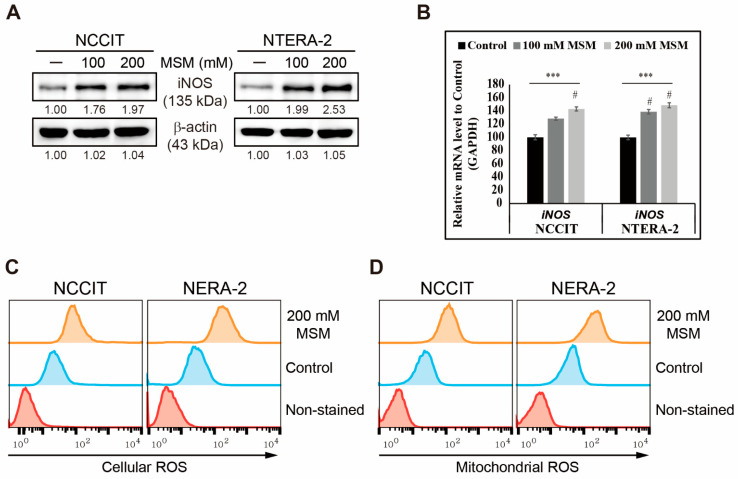
ROS induction in embryonic CSC by MSM. (**A**) Western blotting of NCCIT and NTERA-2 cells with 100 and 200 mM MSM for 48 h shows iNOS protein expression. Expression levels were estimated by densitometry and normalized to β-actin. Data were obtained in triplicate. (**B**) Real-time qPCR analysis showing illustrative expression of the iNOS gene in the NCCIT and NTERA-2 cells. Cp values were normalized to GAPDH mRNA. Controls were set at 100. *** p < 0.001 (ANOVA). # *p* < 0.001 vs. control. (**C**) Flow cytometry of cellular ROS in NCCIT and NTERA-2 cells after treatment with 200 mM MSM for 48 h. The graphical representation shows cells with ROS induction. (**D**) Flow cytometry of mitochondrial ROS by 200 mM MSM in NCCIT and NTERA-2 cells for 48 h. The graphical representation shows cells with mitochondrial ROS induction.

We determined the ability of MSM to inhibit CSC markers and Wnt/β-catenin signaling in NCCIT and NTERA-2 cells. Hence, we assumed that the anticancer activity of MSM might begin by inducing ROS formation. To analyze this, first, we checked the expression pattern of iNOS, which can generate ROS. Our results showed that increasing concentrations of MSM induced the expression levels of iNOS protein in NCCIT and NTERA-2 cells (Figure 2A). To confirm iNOS induction, we analyzed the expression of the iNOS gene in NCCIT and NTERA-2 cells with or without MSM treatment and found a similar result as that in the protein level (Figure 2B). iNOS induction suggested the possible ROS generation by MSM in embryonic CSC. Furthermore, we found that MSM successfully generated cellular ROS (Figure 2C) and mitochondrial ROS (Figure 2D), suggesting that the anticancer activity of MSM is due to ROS generation. We then checked the ability of MSM to induce DNA damage response (DDR) in embryonic CSC. To analyze this, we used a comet assay to determine DNA double-strand breaks and our results showed that MSM induced the DNA double-strand break in NCCIT and NTERA-2 cells (Supplementary Figure S2A). Furthermore, we observed a significant increase in comet length and positive cells in MSM-treated cells compared with nontreated control cells (Supplementary Figure S2B). These results hinted at DDR induction by MSM in embryonic CSC. To confirm this, we analyzed the expression patterns of proteins responsible for DDR and found an elevation in the expression of phosphorylated ATM, ATR, CHK1, CHK2, and BRCA1 by MSM treatment in NCCIT and NTERA-2 cells (Supplementary Figure S2C). These results suggested that ATM or ATR acts as a key regulator in DDR induction by MSM.

### 3.3. MSM Induced Cell Cycle Arrest and TRAIL-Mediated Extrinsic Apoptosis in Embryonic CSC

**Figure 3:**
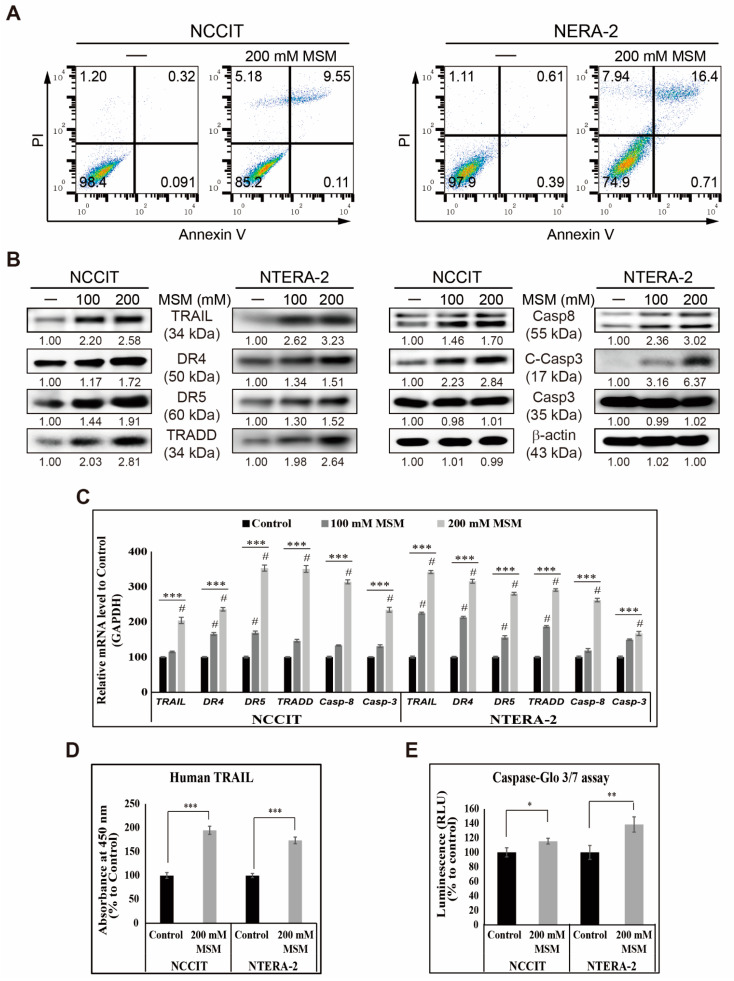
MSM induced extrinsic apoptosis pathway. (**A**) Fluorescein-conjugated annexin V (annexin V-FITC) vs. propidium iodide (PI) staining analysis showed apoptosis induction after treatment with 200 mM MSM for 48 h in NCCIT and NTERA-2 cells. (**B**) Western blotting of NCCIT and NTERA-2 cells with 100 and 200 mM MSM for 48 h showing the expression of TRAIL, DR4, DR5, TRADD, Casp8, Casp3, and C-Casp3 proteins. Expression levels were estimated by densitometry and normalized to β-actin. Data were in triplicate. (**C**) Real-time qPCR analysis showing illustrative expression of genes responsible for the extrinsic apoptosis pathway in NCCIT and NTERA-2 cells with 100 and 200 mM MSM for 48 h. Cp values were normalized to GAPDH mRNA. Controls were set at 100. *** p < 0.001 (*ANOVA* test). # *p* < 0.001 vs. control. (**D**) Human TRAIL assay shows Human TRAIL elevation by 200 mM MSM treatment for 48 h in NCCIT and NERA-2 cells. Controls were set to 100. *** p < 0.001 (Student’s *t*-test). (**E**) Caspase-Glo 3/7 assay showing the enhancement of caspase-3/7 activity by 200-mM MSM treatment for 48 h. Controls were set at 100. * p < 0.05 and ** p < 0.01 (Student’s *t*-test).

On the basis of previous results, we showed that MSM could generate ROS and induce DDR in embryonic CSC. Therefore, we analyzed its role in the cell cycle and apoptosis induction. First, the analysis of cell cycle in embryonic CSC with or without 200 mM MSM and the obtained flow cytometry results showed an arrest in the G0/G1 phase of the cell cycle by MSM (Supplementary Figure S3A). This result indicated that induced DDR results in prolonged cell cycle arrest. To confirm this, we analyzed cell cycle checkpoint genes, *CCND1, CCNE1*, *CDK4*, *CDKN1A*, and *CDKN1B*, in NCCIT and NTERA-2 cells after 48 h of treatment with 200 mM MSM. We found an elevation in the expression levels of tumor suppressor genes, *CDKN1A* and *CDKN1B*, and a decrease in the expression levels of *CCND1*, *CCNE1*, and *CDK4* genes (Supplementary Figure S3B). Protein levels confirmed these results by analyzing the expression pattern of cyclin D1, cyclin E, CDK4, p21, and p27 by Western blotting (Supplementary Figure S3C). These results showed cell cycle arrest by MSM in embryonic CSC and suggested a possible induction of apoptosis by MSM. Thus, we analyzed apoptosis induction by flow cytometry with or without MSM in embryonic CSC, and results showed that MSM induced apoptosis in NCCIT and NTERA-2 cells (Figure 3A). As we found the induction of apoptosis by MSM, we then investigated the apoptosis pathway by checking the key apoptosis regulators, BCL2-associated X (BAX), B-cell lymphoma 2 (BCL-2), and cytochrome C proteins. However, the obtained result was opposite to our expectations as expression levels of BAX and cytochrome C downregulated with increased or unchanged BCL-2 expression in embryonic CSC (Supplementary Figure S4). This forced us to analyze the genes that take part in the extrinsic pathway of apoptosis, and we found an elevation in the expression levels of TRAIL protein along with its receptors, DR4 and DR5, and its downstream mediator, TRADD, and then the upregulation of caspase-8 and -3 precursor proteins (Figure 3B). To confirm the activation of the TRAIL-mediated extrinsic apoptosis pathway by MSM, we analyzed mRNA levels of genes present in the extrinsic apoptosis pathway and found an elevation in the expression levels of all genes (Figure 3C). These results suggested that MSM induced TRAIL-mediated extrinsic apoptosis. We also confirmed the induction of TRAIL by MSM by Human TRAIL assay and observed a significant increase in TRAIL expression (Figure 3D). We then confirmed the extrinsic apoptosis induction by analyzing caspase 3/7 expression and obtained the result indicating a significant elevation of caspase 3/7 by 200-mM MSM in NCCIT and NTERA-2 cells (Figure 3E). Hence, it is confirmed that MSM induced TRAIL-dependent extrinsic apoptosis in embryonic CSC.

### 3.4. Inhibition of Iron Metabolism by MSM in Embryonic CSC

**Figure 4:**
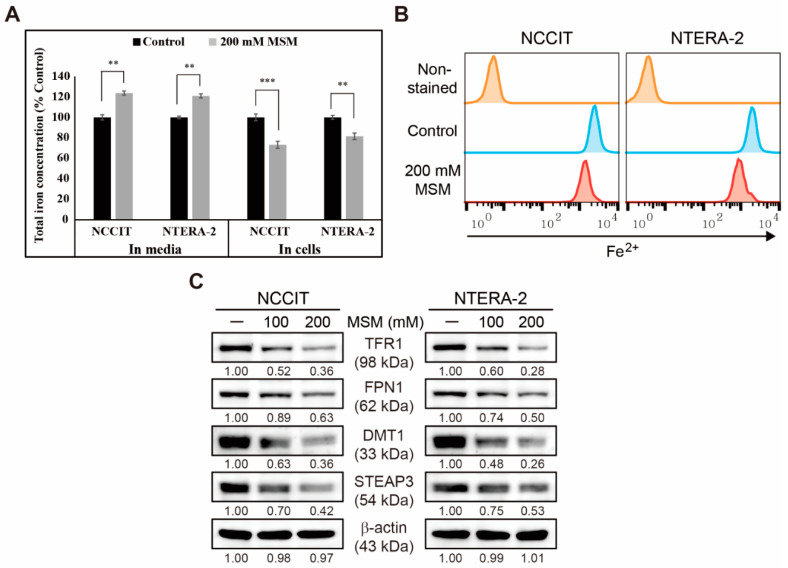
MSM suppressed iron metabolism. (**A**) Iron assay showing total iron concentration in NCCIT and NTERA-2 cells treated with MSM (200 mM) for 48 h. Data were obtained in triplicate. Controls were set at 100. ** p < 0.01 and *** p < 0.001 (Student’s *t*-test). (**B**) Flow cytometry showing the expression of Fe^2+^ in NCCIT and NTERA-2 cells after treatment with MSM (200 mM) for 48 h. The graphical representation shows cells with intracellular Fe^2+^ level. (**C**) Western blotting of TFR1, FPN1, DMT1, and STEAP3 in NCCIT and NTERA-2 cells after treatment with MSM (100 and 200 mM) for 48 h. Expression levels were estimated by densitometry and normalized to β-actin. Data were obtained in triplicate.

We found that MSM can induce apoptosis in embryonic CSCs. Here, we analyzed the mechanistic aspect behind these activities and assumed that iron metabolism might have played a crucial role. First, we estimated iron concentration in cells and media with or without MSM treatment using an Iron Assay Kit (Figure 4A). The results for NCCIT and NTERA-2 cells showed an increase in total iron concentration in media by MSM treatment, whereas, in MSM-treated cells, the concentration of total iron decreased significantly in both cells. This showed the enhancement of iron release from cells to the media. Then, we confirmed this by estimating ferrous ion (Fe^2+^) concentration in NCCIT and NTERA-2 cells after treatment with MSM by flow cytometry, and results suggested a significant decrease in Fe^2+^ ion concentration (Figure 4B). These results indicated an upregulation in iron transport by converting Fe^2+^ to ferric ion (Fe^3+^), which highlighted the conversion of total iron for iron metabolism. To confirm this, we analyzed the expression levels of proteins responsible for iron transport, and the results showed downregulation in the expression levels of transferrin receptor (TFR1) and ferroportin (FPN1) that helps iron intake and outtake (Figure 4C). We also found that MSM suppressed the expression levels of divalent metal transporter 1 (DMT1) and six-transmembrane epithelial antigen of prostate 3 (STEAP3), helping iron conversion. These results suggested the role of iron metabolism in the anticancer activity of MSM against embryonic CSCs.

### 3.5. MSM Upregulated TRAIL Expression by Regulating p38/ERK/p53 Signaling

**Figure 5:**
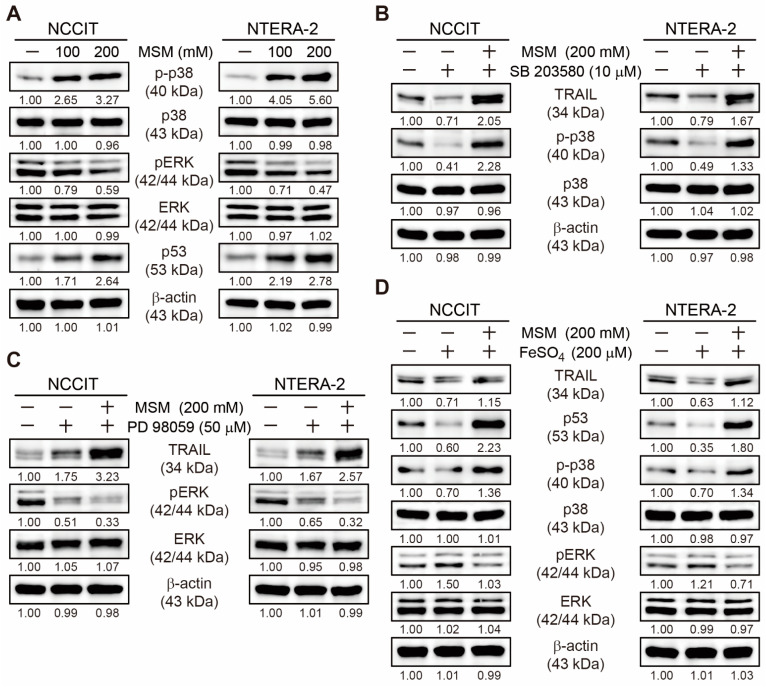
MSM regulated p38/ERK/p53 signaling and TRAIL expression. (**A**) Western blotting of NCCIT and NTERA-2 cells with 100 and 200 mM MSM for 48 h, showing the expression of phospho-p38, p38, phospho-ERK, ERK, and p53 proteins. Expression levels were estimated by densitometry and normalized to β-actin. Data were obtained in triplicate. (**B**) Western blotting of NCCIT and NTERA-2 cells with 10 µM SB 203580 or 200 mM MSM for 48 h, showing the expression of TRAIL, phospho-p38, and p38 proteins. Expression levels were estimated by densitometry and normalized to β-actin. Data were obtained in triplicate. (**C**) Western blotting of NCCIT and NTERA-2 cells with 50 µM PD 98,059 or 200 mM MSM for 48 h, showing the expression of TRAIL, phospho-ERK, and ERK proteins. Expression levels were estimated by densitometry and normalized to β-actin. Data were obtained in triplicate. (**D**) Western blotting of NCCIT and NTERA-2 cells with 200 µM FeSO_4_ or 200 mM MSM for 48 h, showing the expression of TRAIL, phospho-p38, p38, phospho-ERK, ERK, and p53 proteins. Expression levels were estimated by densitometry and normalized to β-actin. Data were obtained in triplicate.

Our previous results showed an upregulation in TRAIL expression by MSM in embryonic CSCs and induced extrinsic apoptosis pathway. Here, we analyzed the molecular signaling for TRAIL upregulation. Additionally, we analyzed the expression levels of phosphorylated p38 and ERK along with p53 and found an elevated expression level of phosphorylated p38 and p53 expression with downregulated expression of phosphorylated ERK protein by MSM in NCCIT and NTERA-2 cells (Figure 5A). This result has hinted at the role of p38/ERK/p53 signaling in TRAIL induction by MSM. To confirm this signaling and its effect on TRAIL expression, first, we used a specific inhibitor of p38 phosphorylation (SB 203580). We found downregulated expression of TRAIL and phosphorylated p38 expression upon SB 203580 treatment with no cytotoxicity effect, and MSM successfully elevated these expressions, suggesting the ability of MSM to induce TRAIL expression by p38 upregulation (Figure 5B). We also confirmed the role of ERK in TRAIL expression using a specific inhibitor of ERK phosphorylation (PD 98,059) with or without MSM in embryonic CSC, and the results showed an increase in the expression of TRAIL upon PD 98,059 treatment with no cytotoxicity effect; MSM addition further increased TRAIL expression in both cells (Figure 5C). These results also indicated that MSM induced TRAIL expression by downregulating ERK expression. Then, we analyzed the role of iron metabolism in TRAIL induction and the p38/ERK/p53 signaling pathway. For this, we used iron sulfate (FeSO_4_) in NCCIT and NTERA-2 cells with or without MSM treatment. The results showed suppression of TRAIL, p53, and phospho-p38 expression levels and an elevation in the expression levels of phospho-ERK by FeSO_4_ treatment with no cytotoxicity effect (Figure 5D). MSM activity successfully reversed these expressions, which suggested the role of iron metabolism in the TRAIL expression by MSM treatment in embryonic CSCs.

### 3.6. MSM Upregulated the Expression of miR-130a and Downregulated miR-221 and miR-222

**Figure 6:**
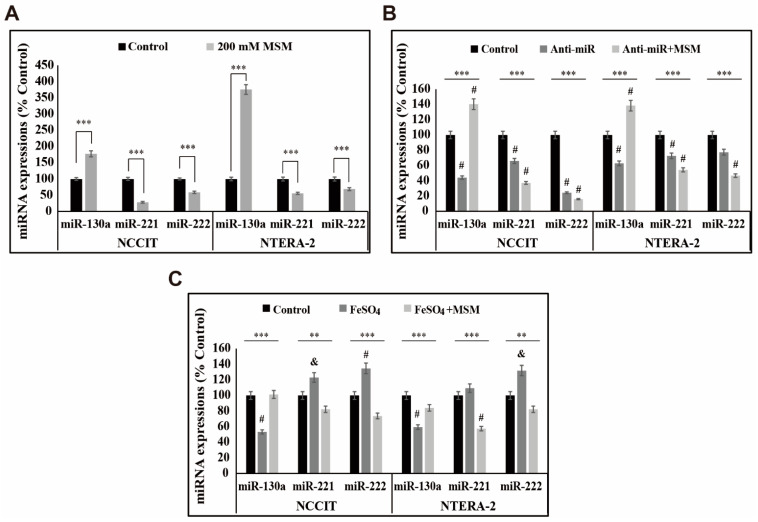
MSM regulated expressions of miR-130a, miR-221, and miR-222. (**A**) Real-time qPCR analysis of miR-130a, miR-221, and miR-222 expressions upon treatment with 200 mM MSM for 48 h in NCCIT and NTERA-2 cells. Representative expression of miR-130a, miR-221, and miR-222 mRNA were shown. Cp values were then normalized to U6 mRNA. Controls were set to 100. *** p < 0.001 (Student’s *t*-test). (**B**) Real-time qPCR of miR-130a, miR-221, and miR-222 expressions upon treatment with 60 pM anti-miR for 24 h, followed by 200 mM MSM for 48 h in NCCIT and NTERA-2 cells. The representative expression of miR-130a, miR-221, and miR-222 mRNA were shown; Cp values were then normalized to U6 mRNA. Controls were set to 100. *** p < 0.001 (ANOVA test). # *p* < 0.001 vs. control. (**C**) Real-time qPCR of miR-130a, miR-221, and miR-222 expressions upon treatment with 200 mM MSM for 48 h, followed by 200 µM FeSO_4_ for 48 h in NCCIT and NTERA-2 cells. The representative expressions of miR-130a, miR-221, and miR-222 mRNA are shown; Cp values were then normalized to U6 mRNA. Controls were set to 100. ** p < 0.01 and *** p < 0.001 (ANOVA test). & *p* < 0.01 vs. control. # *p* < 0.001 vs. control.

**Figure 7:**
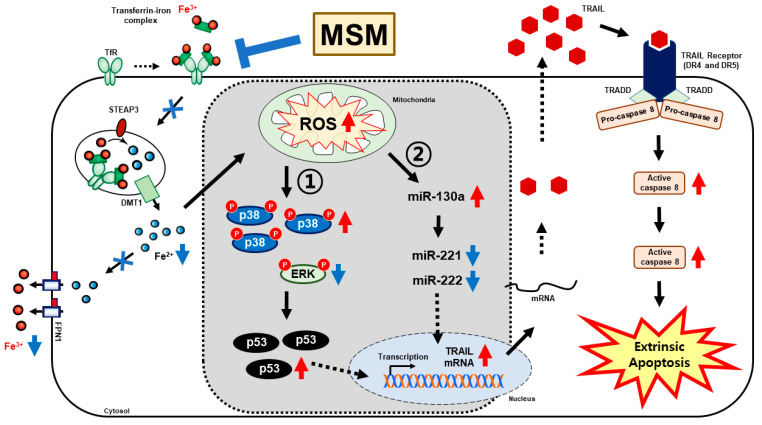
The molecular regulatory mechanism of MSM in the induction of TRAIL-mediated extrinsic apoptosis. MSM inhibited iron metabolism to generate ROS formation, regulating p38/ERK/p53 signaling to induce TRAIL expression. Moreover, MSM enhanced TRAIL expression by upregulating miR-130a and downregulating miR-221 and miR-222 expressions.

We found that iron metabolism and p38/ERK/p53 signaling are vital in inducing TRAIL expression by MSM in embryonic CSCs. Here, we analyzed the role of microRNA in the activity of MSM and the role of iron metabolism in microRNA expression in the presence of MSM. First, we checked the expression levels of miR-130a, miR-221, and miR-222, which play a key role in TRAIL sensitivity. The obtained result from mRNA analysis showed an upregulation in the expression of miR-130a and downregulation in the expression of miR-221 and miR-222 by MSM treatment in NCCIT and NTERA-2 cells (Figure 6A). This may have indicated the role of these microRNAs in TRAIL induction by MSM. To confirm this activity, we used a total microRNA inhibitor anti-miR and then analyzed the expression patterns of these microRNAs in the presence of MSM (Figure 6B). The results showed suppression in the expression of miR-130a by the anti-miR treatment, which was then elevated by MSM treatment, whereas miR-221 and miR-22 expression was further downregulated followed by MSM treatment. These results showed the ability of MSM to regulate these microRNAs. Then, we checked the role of iron metabolism in the expression of these microRNAs using FeSO_4_ treatment with or without MSM. The result showed a decrease in the expression of miR-130a and an increase in the expression of miR-221 and miR-222 by FeSO_4_ treatment, which were successfully reversed by MSM treatment in both cells (Figure 6C). These results also indicated the role of iron metabolism in TRAIL expression by regulating the expression of microRNAs by MSM treatment. Altogether, the molecular mechanism behind the apoptosis induction of MSM begins with the inhibition of iron metabolism, which then induces ROS formation and thereby regulates p38/ERK/p53 signaling, which activates the TRAIL-mediated extrinsic pathway of apoptosis (Figure 7).

## 4. Discussion

Cancer treatment using drugs mainly depends on how they act against cancer cells and prevent cancer recurrence by targeting CSCs. Many chemotherapeutic drugs successfully suppress tumor progression but may have failed to target CSC, resulting in cancer recurrence [35]. This makes the research on natural compounds against cancer interesting as they reduce the side effects so that long-term exposure can help target both cancer cells and CSC. MSM is a natural sulfur-containing compound with a well-known anticancer activity against various cancer cells in vitro and in vivo. Some studies have shown that it can inhibit the tumorsphere formed by colorectal cancer cells [30] and suppress stem cell marker protein expression in breast cancer tissue [36]. Considering the high dose of MSM, it has already been tested as nontoxic in normal cells and animal models, and up to a concentration of 6–8 g/kg/day through oral administration, mice remained healthy and vigorous [37]. Our earlier studies found a non-cytotoxicity effect on bone marrow-derived macrophages isolated from mice by 200-mM MSM [38]. Another cytotoxicity analysis in human monocytes involving THP-1 cells also revealed low or negligible cytotoxicity induced by 200 mM MSM [27]. Therefore, considering MSM to treat embryonic CSCs may provide the best knowledge of its ability to target CSCs without affecting the growth of normal cells. Supporting our hypothesis, MSM successfully inhibited cell viability of embryonic CSC, NCCIT, and NTERA-2 cells, whereas the ability of MSM to target CSC cells depends on how it mediates CSC markers upon treatment. Suppression of CSC markers and Wnt/β-catenin signaling in NCCIT and NTERA-2 cells by MSM suggested that MSM could target cancer cells and CSCs.

A natural compound that can induce DDR that leads to cell cycle arrest and apoptosis in cancer cells could be considered a candidate for further studies. Previous studies have shown that MSM can induce mitochondrial apoptosis against various cancer types [31,32]. ROS generation plays a vital role in the anticancer activity of a natural compound [39]. We found that MSM elevated the expression of iNOS at the transcriptional and translational levels so that iNOS induction leads to ROS generation [24]. As a result, treatment with MSM significantly elevated cellular and mitochondrial ROS, which may signal for anticancer activity. We then demonstrated that MSM could induce DDR by inducing DNA double-strand breaks and inducing the expression levels of ATM or ATR for DDR as these kinases could sense DNA damage and are central regulators in response to DNA damage [40]. This signaling activates p53, leading to the phosphorylation of other substrates such as BRCA1, CHK1, or CHK2 [41,42]. Prolonged DNA damage results in cell cycle arrest for DDR and DNA repair, and p53 takes part in this mechanism [43]. We showed that MSM successfully induced DDR and G0/G1 cell cycle arrest in NCCIT and NTERA-2 cells. The regulation of the expression pattern of cell cycle checkpoints by MSM in NCCIT and NTERA-2 cells also supported cell cycle arrest induction. These results directed toward a possible apoptosis induction in embryonic CSCs by MSM treatment.

The apoptosis pathway was divided into intrinsic and extrinsic pathways so that mitochondria can play a central role in the intrinsic pathway through p53-dependent upregulation of BAX and downregulation of BCL-2 to promote the release of cytochrome C from mitochondria to the cytosol to activate caspase proteins to proceed to apoptosis [44,45]. Flow cytometry results suggested the induction of apoptosis by MSM in NCCIT and NTERA-2 cells. This directed us to analyze the pathway behind apoptosis induction by MSM, and our results were opposite to our expectations, as we observed downregulation in the expression levels of BAX and cytochrome C and upregulation or unchanged expression levels of BCL-2 upon MSM treatment in embryonic CSC, which suggested that MSM does not induce mitochondrial apoptosis. This made us evaluate the death-stimuli mediated extrinsic pathway of apoptosis. Our previous study on NCCIT cells demonstrated the TRAIL-mediated extrinsic apoptosis induction by natural active tannic acid [4]. Hence, we analyzed the role of MSM in the TRAIL-mediated apoptosis pathway in NCCIT and NTERA-2 cells. The extrinsic apoptosis pathway activates when the death ligand, TRAIL, binds its receptor DR4 or DR5 and signals to its adaptor TRADD to activate caspase-8, which activates caspase-3 to promote apoptosis [46,47]. The results were fascinating as MSM induced the expression of TRAIL and its downstream targets DR4, DR5, TRADD, caspase-8, and cleaved caspase-3, which suggested the involvement of extrinsic pathways in MSM-induced apoptosis. We also confirmed this by Human TRAIL and caspase 3/7 assay estimation. Hence, it is evident that MSM induces a TRAIL-mediated extrinsic apoptosis pathway in NCCIT and NTERA-2 embryonic CSC.

The mechanism behind TRAIL-mediated apoptosis induction by MSM was still unclear, which directed us to analyze the role of iron metabolism upon MSM treatment. Iron metabolism can induce ROS generation and promote TRAIL sensitivity by upregulating the expression of p38 and p53 and downregulating the expression of ERK activation [48]. Iron metabolism also plays a crucial role in tumor progression, so that control over iron transport could be a key toward anticancer activity [49]. Adding MSM on NCCIT and NTERA-2 cells showed a decrease in the amount of Fe^2+^ and release of total iron from cells to spent media, suggesting the inhibition of iron transport in NCCIT and NTERA-2 cells by MSM. Molecular analysis of protein responsible for iron transport also provided strong proof for our hypothesis of iron metabolism inhibition by MSM. Hence, ROS generation and inhibition of iron metabolism might take part in the TRAIL induction by MSM. ROS generation inhibits ERK activation and induction of p38 activation and p53 upregulation [50]. The activation of p53 also takes part in extrinsic apoptosis by activating DR4/5 and caspase-8 to promote apoptosis [51]. MSM also enhanced the phosphorylation of p38 and p53 and inhibited the phosphorylation of ERK, suggesting that the inhibition of iron generated ROS, which regulated p38/p53/ERK signaling and TRAIL induction. The role of iron metabolism was also confirmed by the activity of FeSO_4_ affecting TRAIL expression, which was successfully elevated by MSM treatment.

MicroRNAs (miRNAs) take part in various cancer hallmarks by regulating other signaling pathways, inhibiting mRNA translation and promoting mRNA degradation, resulting in the post-transcriptional alteration of gene expression [52]. miRNAs are involved in TRAIL sensitivity in various cancer types for apoptosis induction [53], and among them, miR-130a could induce TRAIL sensitivity by targeting mesenchymal–epithelial transition, also downregulating other miRNAs, miR-221 and miR-222 [54]. Reports also suggested the role of miR-221 and miR-222 to regulate TRAIL resistance and thereby tumorigenicity, and they are overexpressed in many aggressive cancer types [55]. Hence, regulation of miR-130a, mir-221, and miR-222 could hold a key to TRAIL regulation. Treatment with MSM in NCCIT and NTERA-2 cells showed an upregulation in the expression of miR-130a and downregulation in the expressions of miR-221 and miR-222. The regulation of these miRNAs was confirmed using total microRNA inhibitor and MSM treatment, which may have indicated the induction of TRAIL sensitivity in embryonic CSCs. To analyze the role of iron metabolism in these miRNAs’ expression, we used FeSO_4_ to treat NCCIT and NTERA-2 cells and MSM. We found a decrease in the expression of miR-130a and an increase in the expression of miR-221 and miR-222 by FeSO_4_, which was successfully reversed by MSM treatment. As we have previously shown for TRAIL resistance with FeSO_4_ and TRAIL induction by MSM, it is evident that inhibition of iron metabolism plays a key role in these microRNAs to promote TRAIL induction, thereby inducing extrinsic apoptosis pathway.

## 5. Conclusions

This study demonstrated that a natural sulfur-containing compound, MSM, can target CSCs by inhibiting CSC markers and Wnt/β-catenin signaling in NCCIT and NTERA-2 cells. Furthermore, it is also evident that MSM induced TRAIL-mediated extrinsic apoptosis in these embryonic CSCs, and iron metabolism plays a vital role in inducing TRAIL expression through ROS generation, thereby regulating p38/p53/ERK signaling and regulating the expression of miR-130a, miR-221, and miR-222. Altogether, MSM could be considered a candidate for adjuvant chemotherapy as it could suppress cancer recurrence by targeting CSCs.

## Data Availability

The data presented in this study are available on request from the corresponding author. The data are not publicly available due to personal reasons.

## Supplementary Materials

The following is available online at https://www.mdpi.com/article/10.3390/cells10112847/s1, Figure S1. MTT assay showing cell viability inhibition of (A)NCCIT and (B)NTERA-2 cells in different MSM concentrations for 48 h. Data are representative of three independent experiments. Controls were set at 100. **** p <* 0.001 (ANOVA test). # *p* < 0.001 vs. control. Figure S2. MSM induced DDR in embryonic CSC. (A) Comet assay images from fluorescent microscopy at 10× and 40× magnification showing the fragmented DNA migration from the nucleoid body that forms a comet tail in NCCIT and NTERA-2 cells after 48 h treatment with 200 mM MSM. (B) Graphical representation of comet length was analyzed as the fold change versus the control and percentage of comet-positive cells with MSM treatment in embryonic CSCs. **** p* < 0.001 (Student’s *t*-test). (C) Western blotting of NCCIT and NTERA-2 cells treated for 48 h with 100 and 200 mM of MSM showing the expression of phospho-ATM, ATR, CHK1, CHK2, and BRCA1 proteins. Expression levels were estimated by densitometry and normalized to β-actin. Data were obtained in triplicate. Figure S3. MSM induced G0/G1 cell cycle arrest. (A) Flow cytometry using PI staining showing cell cycle distribution in NCCIT and NTERA-2 cells after 48 h treatment with 200 mM MSM. (B) Western blotting of NCCIT and NTERA-2 cells after 48 h treatment with 100 and 200 mM MSM showing the expression of cyclin D1, cyclin E, CDK4, p21, and p27 proteins. Expression levels were estimated by densitometry and normalized to β-actin. Data were obtained in triplicate. (C) RT-qPCR showing expression of cell cycle checkpoint genes. The representative expressions of *CCND1*, *CCNE1*, *CDK4*, *CDKN1A*, and *CDKN1B* mRNA are shown; Cp values were normalized to GAPDH mRNA. Controls were set at 100. **** p* < 0.001 (ANOVA test). # *p* < 0.001 vs. control. Figure S4. Effects of MSM in the intrinsic apoptosis pathway. Western blotting of NCCIT and NTERA-2 cells after 48 h treatment with 100 and 200 mM of MSM showing the expression of BCL-2, BAX, and cytochrome C proteins. Expression levels were estimated by densitometry and normalized to β-actin. Data were obtained in triplicate.
