# Supplementary material for: Iron Metabolism as a Potential Mechanism for Inducing TRAIL-Mediated Extrinsic Apoptosis Using Methylsulfonylmethane in Embryonic Cancer Stem Cells
Source: Cells. 2021 Oct 22;10(11):2847. doi: 10.3390/cells10112847 (PMC8616102; doi:10.3390/cells10112847)
Supplement: Supplementary file 1 [file cells-10-02847-s001.zip › cells-1368529-supplementary.pdf]

cells

Supplementary Material

Iron Metabolism as a Potential Mechanism for Inducing TRAIL-Mediated Extrinsic Apoptosis Using Methylsulfonylmethane in Embryonic Cancer Stem Cells

Nipin Sp [1,†], Dong Young Kang [1,†], Eun Seong Jo [2], Jin-Moo Lee [2,3], and Kyoung-Jin Jang [1,*]

[1] Department of Pathology, Institute of Biomedical Science and Technology, School of Medicine, Konkuk University, Chungju 27478, Korea; nipinsp@konkuk.ac.kr (N.S.); kdy6459@kku.ac.kr (D.Y.K.)
[2] Pharmacological Research Division, National Institute of Food and Drug Safety Evaluation, Osong Health Technology Administration Complex, Cheongju-si 28159, Korea; eses0706@korea.kr
[3] SK Bioscience, Seongnam-si 13493, Korea; elzemy@gmail.com
* Correspondence: jangkj@konkuk.ac.kr; Tel.: +82-2-2030-7839
† These authors contributed equally to this paper.

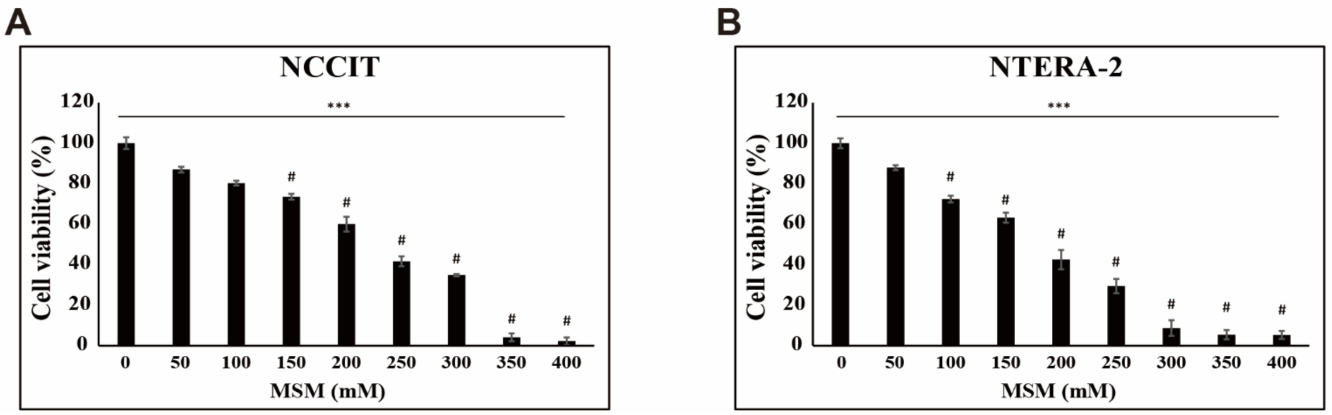

Figure S1. MTT assay showing cell viability inhibition of **(A)** NCCIT and **(B)** NTERA-2 cells in different MSM concentrations for 48 h. Data are representative of three independent experiments. Controls were set at 100. *** $p < 0.001$ (ANOVA test). #$p < 0.001$ vs control.

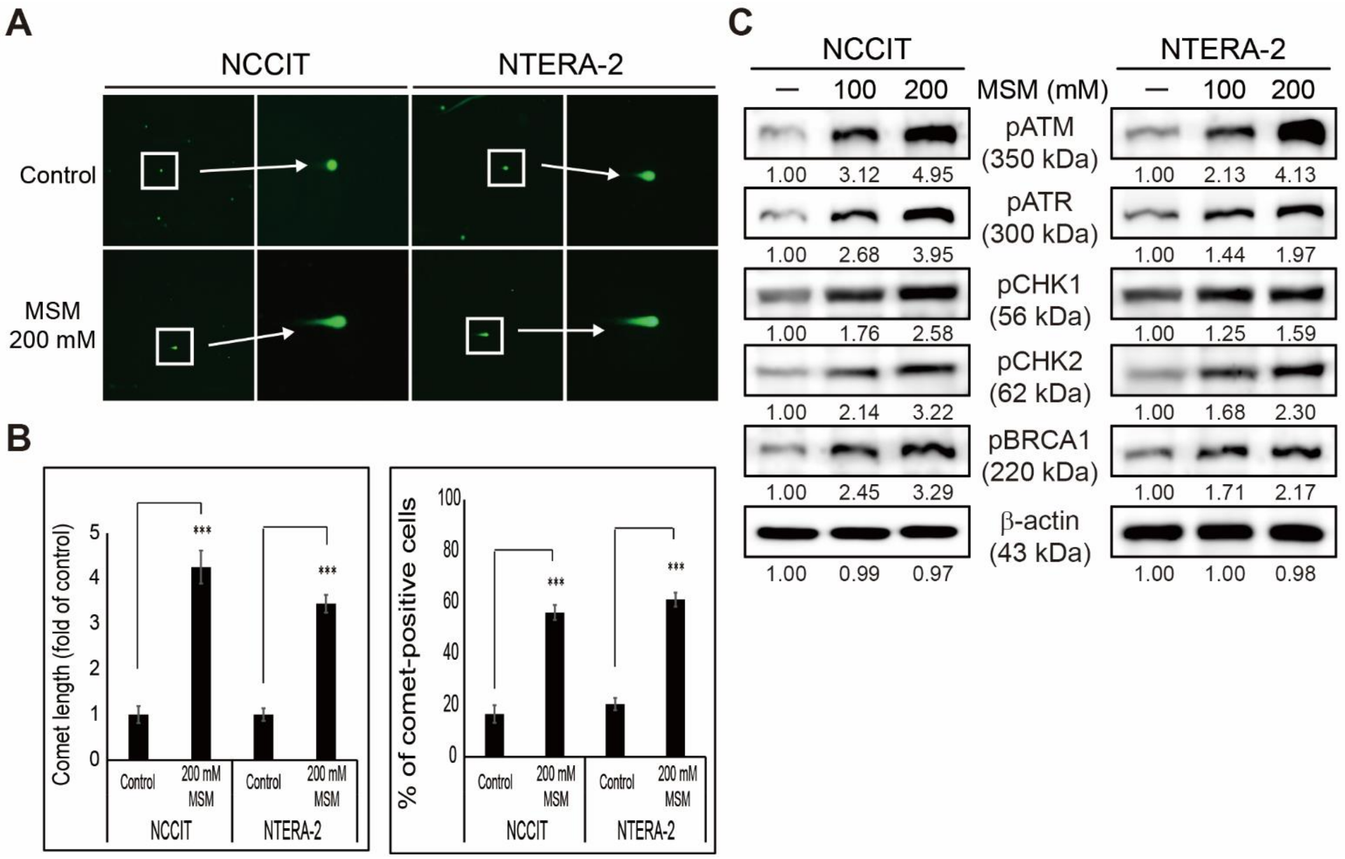

Figure S2. MSM induces DDR in embryonic CSC. **(A)** Comet assay images from fluorescent microscopy at 10× and 40× magnification showing the fragmented DNA migration from the nucleoid body that forms a comet tail in NCCIT and NTERA-2 cells after 48 h treatment with 200-mM MSM. **(B)** Graphical representation of comet length was analyzed as the fold change versus the control and percentage of comet-positive cells with MSM treatment in embryonic CSCs. *** $p < 0.001$ (Student's *t*-test). **(C)** Western blotting of NCCIT and NTERA-2 cells treated for 48 h with 100- and 200-mM of MSM showing the expression of phospho- ATM, ATR, CHK1, CHK2, and BRCA1 proteins. Expression levels were estimated by densitometry and normalized to β-actin. Data were obtained in triplicate.

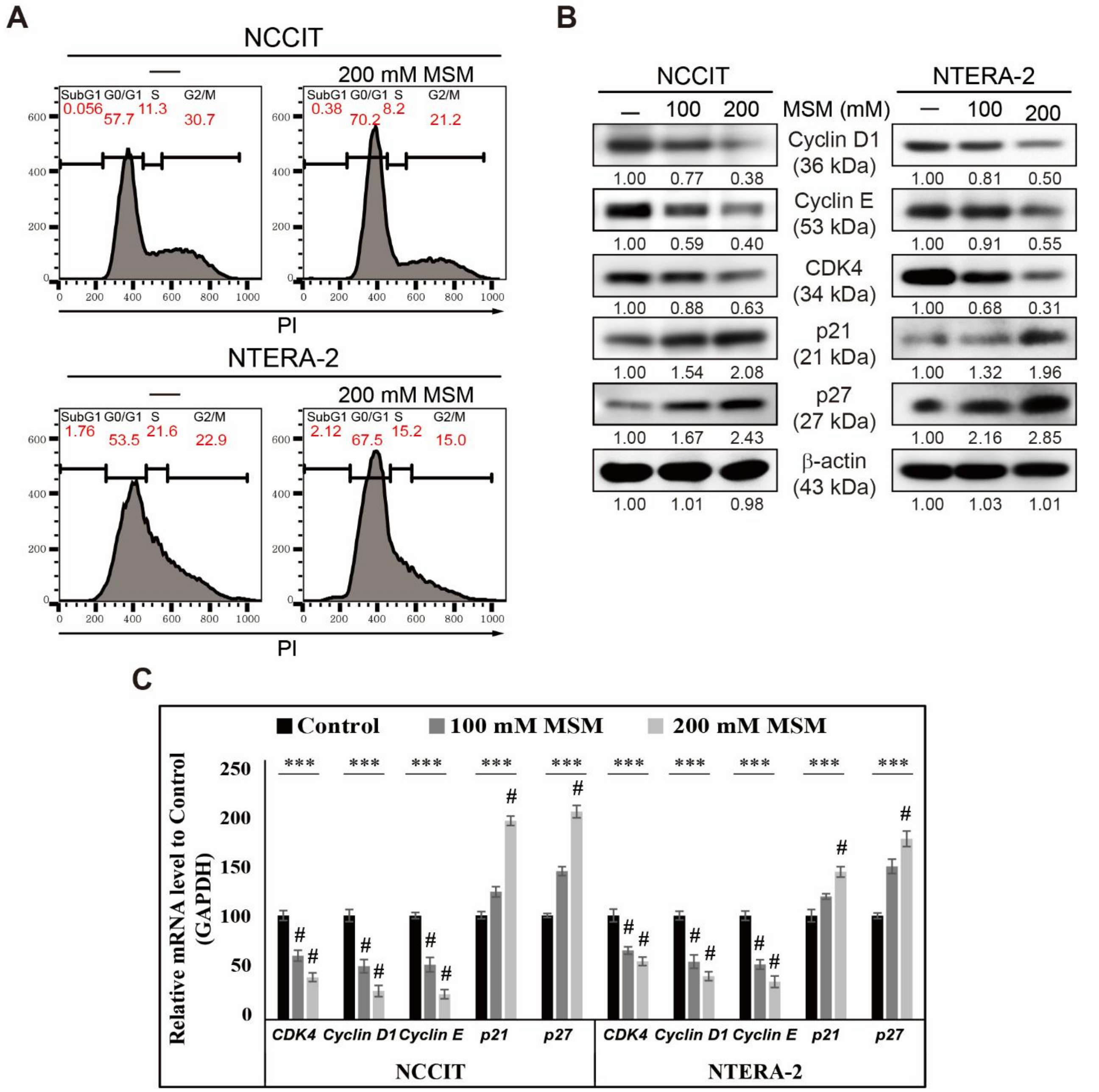

Figure S3. MSM induces G0/G1 cell cycle arrest. **(A)** Flow cytometry using PI staining showing cell cycle distribution in NCCIT and NTERA-2 cells after 48 h treatment with 200-mM MSM. **(B)** Western blotting of NCCIT and NTERA-2 cells after 48 h treatment with 100- and 200-mM MSM showing the expression of cyclin D1, cyclin E, CDK4, p21, and p27 proteins. Expression levels were estimated by densitometry and normalized to β-actin. Data were obtained in triplicate. **(C)** RT-qPCR showing expression of cell cycle checkpoint genes. The representative expressions of *CCND1*, *CCNE1*, *CDK4*, *CDKN1A*, and *CDKN1B* mRNA are shown; Cp values were normalized to GAPDH mRNA. Controls were set at 100. *** $p < 0.001$ (ANOVA test). # $p < 0.001$ *vs*. control.

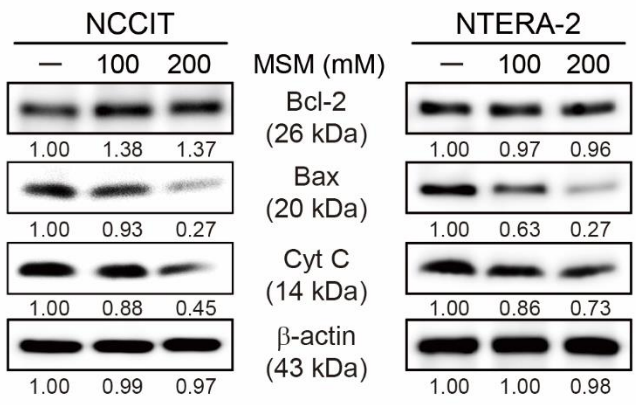

Figure S4. Effects of MSM in the intrinsic apoptosis pathway. Western blotting of NCCIT and NTERA-2 cells after 48 h treatment with 100- and 200-mM of MSM showing the expression of BCL-2, BAX, and cytochrome C proteins. Expression levels were estimated by densitometry and normalized to β-actin. Data were obtained in triplicate.
